# A time-series prediction model of acute myocardial infarction in northern of Iran: the risk of climate change and religious mourning

**DOI:** 10.1186/s12872-021-02372-0

**Published:** 2021-11-23

**Authors:** Hamid Sharif Nia, Ozkan Gorgulu, Navaz Naghavi, Erika Sivarajan Froelicher, Fatemeh Khoshnavay Fomani, Amir Hossein Goudarzian, Saeed Pahlevan Sharif, Roghiyeh Pourkia, Ali Akbar Haghdoost

**Affiliations:** 1 Cardiovascular Research Center, Mazandaran University of Medical Sciences, Sari, Iran; 2 Department of Biostatistics and Medical Informatics, Faculty of Medicine, Ahi Evran University, Kırşehir, Turkey; 3 Faculty of Business and Law, Taylor’s University, Subang Jaya, Selangor Malaysia; 4 Department of Physiological Nursing, School of Nursing, University of California Sand Francisco, San Francisco, CA USA; 5 Department of Epidemiology and Biostatistics, School of Medicine, University of California Sand Francisco, San Francisco, CA USA; 6 School of Nursing and Midwifery, Tehran University of Medical Sciences, Tehran, Iran; 7 Student Research Committee, Mazandaran University of Medical Sciences, Sari, Iran; 8 Faculty of Business and Law, Taylor’s University Malaysia, Subang Jaya, Malaysia; 9 Department of Cardiology, Cardiovascular Research Center, Babol University of Medical Sciences, Babol, Iran; 10 Research Center for Modeling in Health, Institute for Futures Studies in Health, Kerman University of Medical Sciences, Kerman, Iran

**Keywords:** Weather, Acute myocardial infarction, Time series, Iran

## Abstract

**Background:**

Although various studies have been conducted on the effects of seasonal climate changes or emotional variables on the risk of AMI, many of them have limitations to determine the predictable model. The currents study is conducted to assess the effects of meteorological and emotional variables on the incidence and epidemiological occurrence of acute myocardial infarction (AMI) in Sari (capital of Mazandaran, Iran) during 2011–2018.

**Methods:**

In this study, a time series analysis was used to determine the variation of variables over time. All series were seasonally adjusted and Poisson regression analysis was performed. In the analysis of meteorological data and emotional distress due to religious mourning events, the best results were obtained by autoregressive moving average (ARMA) (5,5) model.

**Results:**

It was determined that average temperature, sunshine, and rain variables had a significant effect on death. A total of 2375 AMI’s were enrolled. Average temperate (°C) and sunshine hours a day (h/day) had a statistically significant relationship with the number of AMI’s (β = 0.011, *P* = 0.014). For every extra degree of temperature increase, the risk of AMI rose [OR = 1.011 (95%CI 1.00, 1.02)]. For every extra hour of sunshine, a day a statistically significant increase [OR = 1.02 (95% CI 1.01, 1.04)] in AMI risk occurred (β = 0.025, *P* = 0.001). Religious mourning events increase the risk of AMI 1.05 times more. The other independent variables have no significant effects on AMI’s (*P* > 0.05).

**Conclusion:**

Results demonstrate that sunshine hours and the average temperature had a significant effect on the risk of AMI. Moreover, emotional distress due to religious morning events increases AMI. More specific research on this topic is recommended.

## Background

Myocardial infarction (MI) as a result of acute coronary syndrome, refers to the permanent and irreversible cellular death of myocardium secondary to loss of blood flow and prolonged lack of oxygen supply (ischemia) [1]. ST-segment elevation myocardial infarction (STEMI), and non-ST-segment elevation are two types of MI. While the cause of mismatch between myocardial oxygen demand and myocardial oxygen consumption in STEMI is nearly always coronary plaque rupture resulting in thrombosis formation occluding a coronary artery, there are several potential causes of this mismatch in NSTEMI [2]. These life-threatening condition may appear suddenly and without any earlier symptoms, or after several angina attacks (chest pain) [3]. While only less than 10% of all deaths worldwide was attributed to cardiovascular disease at the beginning of the twentieth century, the current prevalence of this disease increased as the passage of the time and the impact of the industrial revolution on the social and economic dimensions of life [4]. AMI more than any other disease not only causes deaths but also results in disabilities and economic burdens [5].

While MI is most prevalent in older adults, it is also present in younger people [3]. The prevalence of this disease is increasing in developing countries; which means that 82% of the 16 million worldwide deaths related to AMI results in disability due to this disease [4]. Furthermore, AMI has increased twelve times in women and almost fourteen times in men in developing countries [6]. According to the Iranian Ministry of Health and Medical Education, 39.3% of annual death is due to heart disease and of these, 19.5% result from AMI [1]. The high incidence of AMI in developing countries is associated with less physical activity, weight gain, tobacco use, occupational and mental stress, and low health literacy [7].

Studies over the past decades have addressed various mechanisms and leading factors of AMI [8–10]. Risk factors affecting the occurrence of AMI are categorized into non-modifiable (age, gender, family history, weather) and modifiable (smoking, physical activity, psychological tension) factors [11–14]. Among non-modifiable factors, weather condition has not received much attention in the literature until recently. Few studies have been conducted for more than 50 years around the world [15] to investigate if weather conditions affect AMI. The non-modifiable factors described as “triggers” are defined as external stimuli triggering the internal mechanism that led to AMI. While acute triggers are classified into four categories of emotional, environmental, physical and chemical, in this study environmental and specially meteorological triggers have been further analyzed [16, 17].

Studies revealed that meteorological parameters and seasonal climate changes play an important role in the incidence of AMI [18, 19], so that approximately 4% of the incidence of AMI is associated with various types of these parameters [18–20]. Weather conditions such as average temperature, average humidity, wind speed, and wind pressure are identified parameters associated with AMI [7, 21, 22]. While the majority of studies revealed that the AMI incidence increase during winter and spring [15, 23–25], some studies showed that AMI occurs in the summer when the temperature and humidity are high and the atmospheric pressure is low [26]. It seems that physiological stressors such as sympathetic activation, high blood coagulation, and infection in cold climates (such as influenza and air pollution) are associated with AMI [24, 27, 28]. Hemodynamic changes are also intensified in the winter. Moreover, an increased immune response, high blood pressure, immobility and the risk of respiratory infections associated with low temperatures in winter can affect the incidence of AMI [29, 30]. The study findings by Versaci et al. [31] revealed that investigating the impact of climate change on coronary atherothrombotic events should be undertaken by considering season-specific patterns and the trends in climate features in the days preceding the acute event. Accordingly, a higher risk of acute myocardial infarction is expected with lower temperatures, lower minimum atmospheric pressure (ATM), and lower rainfall in Winter, greater changes in ATM and greater humidity in Spring, and higher temperatures in Summer [31].

The extensive literature has supported the exposition that pollutant agents such as fine particles (particulate particles with a diameter ≤ μm [PM2.5]) would increase the cardiovascular morbidity and mortality rate [32–34]. The yielded result from ‘Global Burden of Disease’ has ranked environmental pollutants among the top 10 determinants of cardiovascular disease out of the investigated 67 risk factors. The results have also highlighted the impact of PM2.5 on the ischemic cardiovascular disease [35].

Although various studies have been conducted on the effects of climate variables and seasonal climate changes on the risk of AMI, many of them have limitations to determine the predictable model. Epidemiological models can predict the prevalence of AMI before its onset using a combination of the variables. There is a balance between the accuracy and timeliness of the predictions made with this method; the closer we get to the time of the occurrence of AMI, the more accurate the predictions are, but less time is available for the necessary action [36]. It should be considered that the relationship between the predictive variables of AMI is very complex. Moreover, the biological, statistical, and mathematical models have been faced by some limitations due to their complexity for clinical applications.

Emotional triggers as the influencing factor of AMI incidence include anger, anxiety or depression secondary to hearing unexpected news, work-related stress, and war. Since triggers reduce sleep and appetite as well as increase in cortisol level, they are considered as factors contributing to cardiovascular disease [37, 38]. Steptoe et al. [39] showed that chronic stress at work, and in private life increases the occurrence of heart disease by 40–50%. Similarly, admission rates for AMI increased after the death of a loved ones or during the bereavement periods [40, 41]. Unlike anger or anxiety, the effect of bereavement is thought to persist for weeks and months [42]. While acknowledging the linkage between psychological stress and AMI. Brotman, Golden, Wittstein [43] discussed that the risk of AMI due to catastrophic emotional events is more prevalent in patients with pre-existing heart disease [43].

Iran is a country of four seasons and the temperature variation from summer to winter is significant. Iran seems an interesting context for the topic of this study as it is one of the only countries in the world that has the complete four seasons. A large part of the country suffers great extremes of heat and cold between summer and winter. Rainfall is mainly confined to winter and spring. Summers are hot with virtually continuous sunshine. Highly humid weather in summer especially along the Caspian Sea in the north and Persian Gulf increases the danger of heat exhaustion. Moreover, the official religion in Iran is Islam and the ruling regime that has tried to inject Islamic laws into every aspect of Iranian life. Therefore, the religious functions, and rituals are held every year in the form of mass folk flow and movement.

Considering the increased prevalence of AMI among in not only the elderly but also the younger generation and its impact on the quality of life requires a better understanding of all risk factors. Furthermore, religion and culture are intertwined with the lifestyle of Iranian people, and religious beliefs, functions, and rituals have an important impact on the lives of Iranian Muslims. The mournful religious activities, as well as nation-wide sadness during some months, are well-known as many people publicly display their mourning. The main objective of this study is to provide a prediction model for the occurrence of AMI using meteorological variables and emotional triggers.

## Methods

### Data

The data of AMI patients was collected from the Mazandaran Province Heart Center, Iran located at the following coordinates (36.369 N, 52.270 W) because it offers the most comprehensive data of AMI patients in Iran [44]. Census sampling method was used between 25/03/2011 and 20/03/2018.

The following were considered as the definitive diagnostic criteria were: (1) existence of cardiac enzymes (CK or CK-MB) above the normal range; (2) ST-segment elevation or depression of more than 1; (3) abnormal Q waves; and (4) manifestation of Troponin enzyme in the blood [1].

The following variables were extracted: gender, the day, month, year and time of hospital admission and recovery situation. Also, weather variables were included daily temperature (Celsius) changes (minimum, maximum, and average), wind speed (meters per second) and its direction, rainfall (day), daily evaporation rate (mm), number of sunny days, and relative humidity (percent) between March 2011 to March 2018 were provided by the Meteorological Organization of Iran. Iran’s four climate seasons are: spring (April to June), summer (July to September), autumn (September to December) and winter (January to March). In this study, TRAMO/SEATS technique was used for all series in order to seasonally adjustment. The process of removing the seasonal component from a time series is known as deseasonalization or seasonal adjustment, and the time series thus obtained is called the deseasonalized, or seasonally adjusted, time series. TRAMO/SEATS method, developed by Gomez and Maravall [45]. TRAMO initially models the series with AR (1) and ARMA (1, 1) to determine the periodic and seasonal difference levels. The appropriate seasonal or non-seasonal ARMA model is selected according to BIC criterion; TRAMO also automatically identifies outliers and calculates other regression variables. Then, TRAMO passes the linearized series to SEATS, where the actual decomposition is done. In SEATS, first the spectral density function of the estimated model is decomposed into the spectral density function of the unobserved components, which are assumed orthogonal. SEATS then estimates the parameters of the two components (trend-cycle and seasonally adjusted component) [46, 47].

The emotional triggers in this study are the religious events in Iran. The ‘event’ variable in the model refers to mournful religious events in Iran.

### Statistical method

Box–Jenkins (B–J) estimation models, one of the time series analysis methods, are widely used for modeling and predictions in the field of health and in different applied sciences. B–J method is a linear structure model that traces all the past values of the variable and its stochastic components to predict the values for all the future periods. Prior to have the details of B–J method of forecasting we need to see how a time series data of a particular variable is generated. There are three processes behind the cohort of a time series data.

1. AR (autoregressive) Process: Past values of the variable and error term generate the data
2. MA (moving average) Process: Only the errors or the disturbance term generate the data
3. ARMA (autoregressive and moving average) Process: Data is generated by the combination of AR and MA processes

Sometimes it is taken as ARIMA model where ‘I’ stands for the order of Integration of the series or how many differencing is done for making the time series of the variable to Stationary [48].

B–J methods are known as Autoregressive (AR), Moving Average (MA), Autoregressive Moving Average (ARMA) and Autoregressive Integrated Moving Average (ARIMA) [49, 50]. AR(p), MA (q) and ARMA(p,q) when the process is stationary in the time series that is being examined; ARIMA(p,d,q) can perform applications when it is not stationary [48].

#### Autoregressive model-AR(p)

The mathematical representation of AR model is given in Eq. 1. In Eq. 1, p is the number of autoregressive components. If there is no relationship between neighboring observation values, p value is zero. An AR(p) process is one where the current or present period’s value of a variable ‘y’ depends on only the past values plus an error term. If there are ‘p’ order in the process i.e. current value of y depends on the ‘p’ order of past (e.g. t − 1, t − 2, etc.) values and an error term of the current period then the AR(p) can be written as:

1 $$y_{t} = \mu + \varphi_{1} y_{t - 1} + \varphi_{2} y_{t - 2} + \varphi_{3} y_{t - 3} + \cdots + \varphi_{p} y_{t - p} + u_{t} = \mu + \sum \varphi_{i} y_{t - i} + t_{t}$$

In Eq. 1, μ is constant term, u_t_ is White nose error term with zero mean, constant variance and zero auto-covariance [48].

#### Moving average-MA (q)

An MA(q) process, on the other hand, is the linear combination of all the q terms of the past values of the white noise terms depending on time. When there is no moving average component, q is zero. It is a white noise process in which the current value of y_t_ depends on the current value of the white noise error term (u_t_) and all past values of the error terms. Because all the errors are white noise, so, an MA process is necessarily a stationary process. It is true further because it is the linear combination of all plus and minus values of the errors which hover around the value zero. The mathematical representation of MA model is given in Eq. 2.

2 $$y_{t} = u_{t} + \theta_{1} u_{t - 1} + \theta_{2} u_{t - 2} + \theta_{3} u_{t - 3} + \ldots + \theta_{q} u_{t - q} = u_{t} + \sum \theta_{i} u_{t - i}$$

An AR process is stationary if the characteristic root lies outside the unit circle or having values > 1. If it is so then then *ϕ* becomes less than 1. This means the condition ϕ < 1 lead to the values lying inside the unit circle representing stationarity of the AR process, the model is thus having stability property. The AR coefficients should then be less than unity or they should lie within the unit circle [48].

#### Autoregressive moving average-ARMA(p,q)

An ARIMA(p,q) process is the combination of AR and MA process, I being the order of integration which can be represented by ‘d’, number of differencing to convert the series from nonstationary to stationary. The model for ARMA(p,d,q) can then be written as,

3 $$y_{t} = \mu + \varphi_{1} y_{t - 1} + \varphi_{2} y_{t - 2} + \varphi_{3} y_{t - 3} + \cdots + \varphi_{p} y_{t - p} + u_{t} + \theta_{1} u_{t - 1} + \theta_{2} u_{t - 2} + \theta_{3} u_{t - 3} + \cdots + \theta_{q} u_{t - q}$$

Using lag operatör, we have

4 $$\left( {1 - \varphi_{1} L - \varphi_{2} L^{2} - \varphi_{3} L^{3} - \varphi_{4} L^{4} - \ldots \varphi_{p} L^{p} } \right)y_{t} = \mu + \left( {1 + \theta_{1} L + \theta_{2} L^{2} + \theta_{3} L^{3} + \ldots \theta_{q} L^{q} } \right)u_{t} or, \varphi \left( L \right)y_{t} = \mu + \theta \left( L \right)u_{t}$$

This relation stands (Eq. 4) for invertibility between the AR and MA process which means AR and MA processes can be made invertible from one to another [48, 51].

In this study, an AMI event was analyzed by using Autoregressive Moving Average (ARMA) model because of the time series’ structure is stationary. In order to determine whether the time series is stationary or not, a unit root test was applied to the data. Augmented Dickey Fuller (ADF) unit root test and Philips and Perron (PP) unit root test were used [52]. In the analysis, the optimal structure of ARMA model was discussed in various combinations of p and q values and the appropriate time series model was determined, the number of future AMI’s was estimated. The Ordinary Least Square (OLS) methods were used to estimate the parameters. Akaike Information Criterion (AIC) was used to evaluate the performance of the models [51, 53, 54].

#### Poisson regression analysis

The count model was applied to analyze the occurrence of AMI. Poisson Regression analysis is a count model method analysis that describes the relationship between independent (reason) variables and the dependent (response) variable obtained by counting. The underlying structure in the Poisson regression analysis is that the Y response variable is the discrete independent Poisson random variable. It is one of the methods shown as an alternative to classical linear regression analysis because of the failure of normality assumption [55].

The Poisson distribution has the following moments,

5 $${\text{E}}\left( {\text{X}} \right) = \upmu \;{\text{and}}\;{\text{Var}}({\text{X}}) = \upmu$$

The Poisson distribution assumes variance equal to the mean and, hence, it has limitations when dealing with overdispersal data, i.e., when the sample variance exceeds the sample mean. The opposite is called the under-dispersion problem (when the sample mean exceeds the sample variance). In this case negative binomial Poisson regression analysis is used [56].

The Poisson regression model determined the probability that the regressed variable (Y) occurs at a specific time interval.

The probability is modeled as:

$$P\left( {Y = y} \right) = \frac{{e^{ - \mu } \mu^{y} }}{y!}$$

where Y: dependent variable; $\mu$ = mean parameter [56];

In our study, the dependent variable was number of patients with AMI per day. Explanatory variables were event (mournful religious activities), average temperature, average humidity, sunshine, raining, daily evaporation rate, wind direction, wind speed. The mean of dependent variable (number of patients with AMI) was 3.62 per day, which was similar to the variance result (3.64). If the mean and variance of the dependent variables are equal, the Poisson regression model would best fit the dependent variable of interest. There is not over dispersion and under dispersion problem in our data set. So, we used to Poisson regression analyze. In order to determine the most suitable model, the goodness of fit coefficients was taken into consideration. The analysis was performed with MATLAB R2016a and EViews 9.0.

## Results

The sample were 2375 patients with AMI. Table 1 shows the descriptive statistics for the study variables.

**Table 1:** Descriptive statistics for the study variable from 01 January to 16 March, 2013–2015

Variables	Mean (SD)	Median (IQR:25th percentile–75th percentile)
Average temperature	18.19 (7.54)	18 (11.50–25.80)
Average humidity	77.50 (9.04)	77.5 (72–83.50)
Sunshine hours a day	5.71 (3.96)	6 (1.55–9.00)
Raining	1.82 (6.75)	0 (0.0–0.20)
Daily evaporation rate	3.18 (2.36)	2.6 (1.10–5.20)
Wind direction	201.34 (128.66)	260 (40–300)
Wind speed	4.31 (2.08)	4.0 (3.0–5.0)

**Fig. 1:**
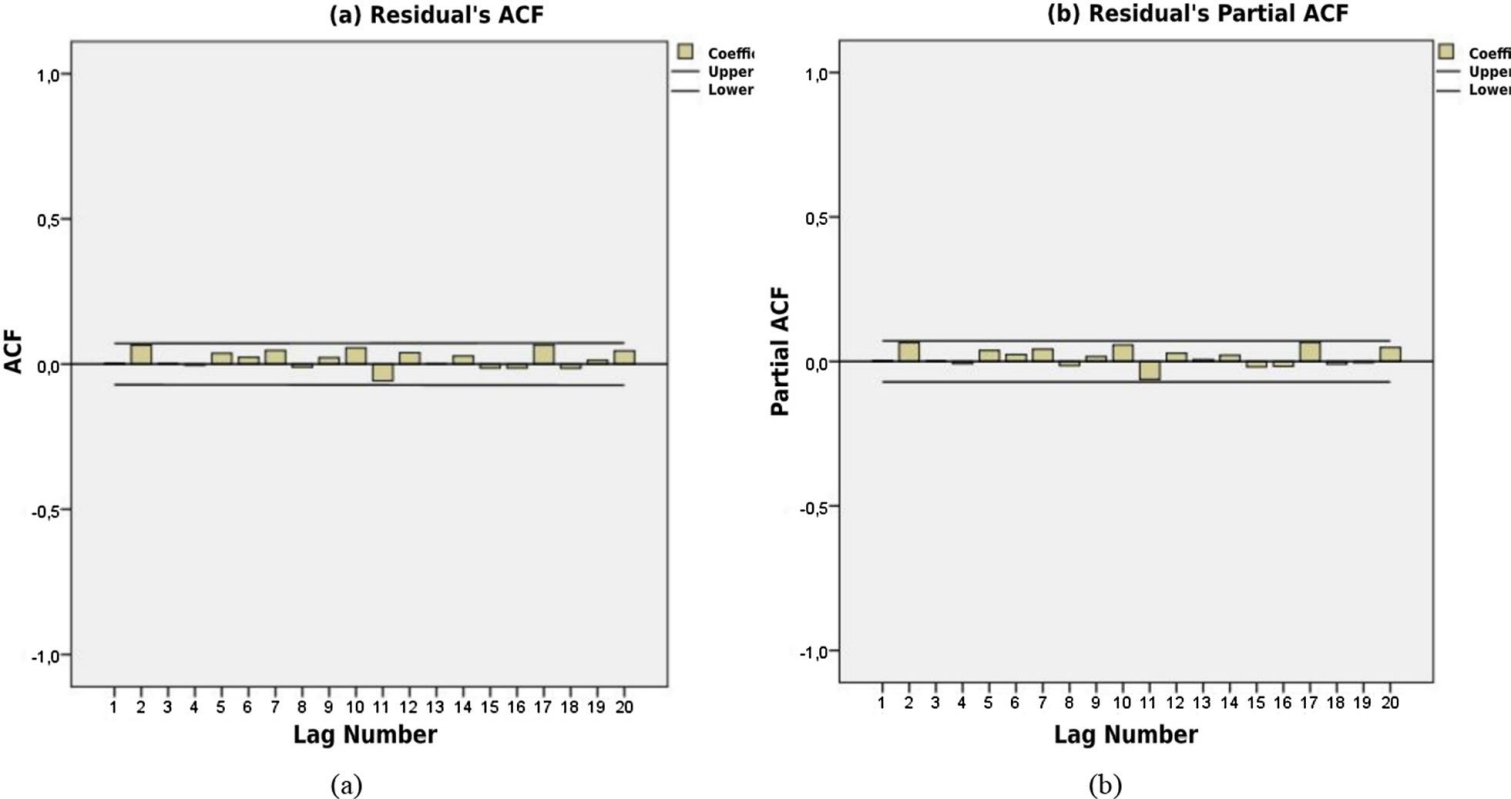
ACF (**a**) ve PACF (**b**) figures

In order to perform the analysis of ARMA model, the correlograms of time series were examined with ACF and PACF graphs. Figure 1a and b show the ACF and PACF correlograms for the examined time series, respectively. Boundary lines around functions represent 95% confidence limits. There are no values exceeding the confidence limit in correlograms. In addition, the statistical significance of the autocorrelation coefficients was tested with the Ljung–Box Q statistic. Correlogram graphs and Q statistic values show that the series is stationary (Table 2).

**Table 2:** AC, PAC and Q-stat results for heart attack series

Lag	AC	PAC	Q-Stat.	Prob.
1	0.034	0.034	0.9250	0.336
2	0.002	0.001	0.9298	0.628
3	− 0.004	− 0.004	0.9415	0.815
4	− 0.030	− 0.029	1.6504	0.800
5	− 0.041	− 0.039	3.0091	0.699
6	− 0.024	− 0.021	3.4722	0.748
7	0.006	0.007	3.5007	0.835
8	0.016	0.015	3.7093	0.882
9	0.016	0.012	3.9054	0.918
10	− 0.023	− 0.027	4.3391	0.931
11	0.041	0.042	5.7223	0.891
12	0.006	0.004	5.7505	0.928
13	− 0.030	− 0.029	6.5013	0.926
14	− 0.023	− 0.021	6.9458	0.937
15	0.026	0.029	7.4952	0.942
16	0.017	0.017	7.7295	0.957
17	0.058	0.058	10.482	0.882
18	0.024	0.017	10.966	0.896
19	0.022	0.018	11.353	0.911
20	− 0.014	− 0.015	11.524	0.931

Augmented Dickey Fuller (ADF) unit root test and Philips and Perron (PP) unit root test were used to determine whether the time series is stationary or not. The results of the ADF and PP tests are given in Table 3. Analysis results show that the time series does not contain unit roots and has a stationary structure.

**Table 3:** ADF and PP unit root tests

Variables	ADF test statistics	PP test statistics
AMI	− 28.22	− 28.19
Average temperature	− 14.34	− 14.25
Average humidity	− 16.61	− 16.66
Raining	− 23.74	− 23.73
Sunshine hours a day	− 12.32	− 17.36
Daily evaporation rate	− 3.53	− 10.17
Wind direction	− 25.93	− 25.94
Wind speed	− 24.75	− 24.78
*Critical values*		
1%	− 3.43	− 3.44
5%	− 2.86	− 2.86
10%	− 2.56	− 2.57

The maximum lag length is taken as 20

Various AR, MA and ARMA models were analyzed with different p and q degrees in order to model the number of heart attacks with time series. Since the correlogram graphs did not provide definite information about AR, MA and ARMA, the models were analyzed one by one until p and q values were in sixth degree. Table 4 shows the AIC results calculated with different p and q values. Accordingly, the model with the lowest AIC value determined the most suitable ARMA structure for the time series examined. Results show that ARMA (5,5) has the lowest AIC value in parameter combinations. The lowest calculated AIC value was calculated as 4.6058. Table 5 shows AR and MA parameter estimation values, standard errors, t-stats and probability values estimated by ARMA (5,5) model. Related numerical findings are given in Table 5.

**Table 4:** AIC results for ARMA model

ARMA	q					
p	1	2	3	4	5	6
1	4.6096	4.6058	4.6248	4.6328	4.6266	4.6157
2	4.6178	4.6100	4.6126	4.6315	4.6347	4.6342
3	4.6204	4.6187	4.6157	4.4.6219	4.6114	4.6205
4	4.6261	4.6257	4.6056	4.6159	4.6151	4.6215
5	4.6149	4.6216	4.6229	4.6059	4.6058	4.6071
6	4.6162	4.6209	4.6376	4.6222	4.6083	4.6024

**Table 5:** ARMA model parameter estimations

Variable	Coefficient	Std. Error	t-Statistic	Prob.
AR(1)	0.637889	0.006320	100.9265	0.0000
AR(2)	− 0.231091	0.006209	− 37.21658	0.0000
AR(3)	0.246695	0.005654	43.63024	0.0000
AR(4)	− 0.638374	0.006419	− 99.44966	0.0000
AR(5)	0.983826	0.006121	160.7385	0.0000
MA(1)	− 0.629692	0.005422	− 116.1446	0.0000
MA(2)	0.238851	0.005928	40.29171	0.0000
MA(3)	− 0.241633	0.005693	− 42.44279	0.0000
MA(4)	0.628322	0.005260	119.4531	0.0000
MA(5)	− 0.986092	0.004266	− 231.1485	0.0000
R-squared	0.029068	Mean dependent var		2.952500
Adjusted R-squared	0.018007	S.D. dependent var		2.427470
S.E. of regression	2.405515	Akaike info criterion		4.605826
Sum squared resid	4571.336	Schwarz criterion		4.664384
Log likelihood	− 1832.330	Hannan–Quinn criter		4.628321
Durbin–Watson stat	1.931567			
Inverted AR roots	1.00	.51 − .85i	.51 + .85i	− .69 − .72i
	− .69 + .72i			
Inverted MA roots	1.00	.50 + .86i	.50 − .86i	− .69 + .72i
	− .69 − .72i			

**Fig. 2:**
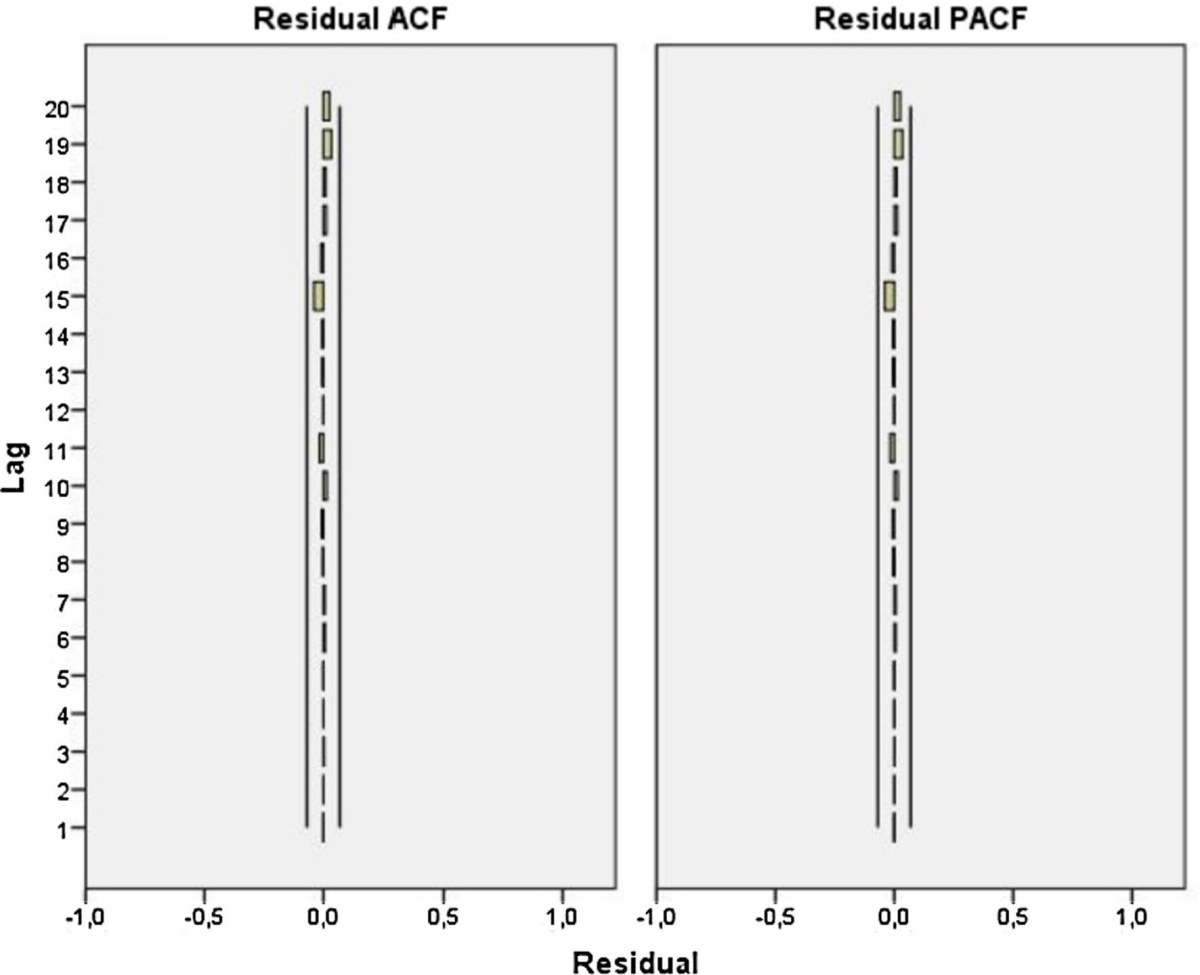
Residual’s ACF (**a**) and PACF (**b**) graphs

Autocorrelation and heteroscedasticity of residuals were examined in the process of examining whether the analyzed model was appropriate for the data set. The results of ARMA (5,5) model were investigated with Box–Ljung Q test by using model residuals. Here, H_0_ hypothesis: Model is appropriate; H_1_ hypothesis: Model is inappropriate. Ljung–Box Q test results show that ARMA (5,5) model is appropriate. For model residuals, ACF and PACF correlograms are shown in Fig. 2. As can be seen in the graphs, only a single value is out of the confidence limit with a relatively small margin.

The heteroscedasticity of residuals was examined by the ARCH test. According to ARCH results, there is no heteroscedasticity in the examined data set. As can be seen in the Table 6, the F-stat value was found to be 0.400478, prob. F (1.797) and Prob. Chi-Square 0.5264.

**Table 6:** ARCH results for ARMA

F-statistic	0.400478	Prob. F(1.797)	0.5270
Obs*R-squared	0.401281	Prob. Chi-Square(1)	0.5264

Omnibus test value is significant (Likelihood Ratio Chi-Square = 24.082, *P* < 0.001). In this case, the Poisson regression model is statistically significant. Based on Goodness of fit value (Deviance, scaled deviance, AIC, AICC, BIC) estimates, the Poisson regression model fit the data (Table7).

**Table 7:** Goodness of fit value of Poisson regression model

	Value	Value/df
Deviance	783.67	1.213
Pearson chi-square	821.34	1.271
Akaike’s information criterion (AIC)	2769.85	
Finite sample corrected AIC (AICC)	2770.13	
Bayesian information criterion (BIC)	2810.21	

Table 8 shows the results from Poisson regression analysis. The model was run to estimate the relative risk (RR), which measures the rate at which the number of patients with AMI per day. Table 8 illustrates the RRs estimated across the explanatory variables. Average Temperature variable has significant effect on the AMI (β = 0.011, *P* = 0.014). For every extra degree of temperature increase 1.011 (95%CI 1.002–1.019) times risk of AMI. For every extra an hour sunshine 1.025 (95% CI 1.010–1.040) times more AMI risk was occurred, a statistically significant result, (β = 0.025, *P* = 0.001). The risk of AMI will be 0.948 times greater for Event (0) meaning the mournful religious activities can increase the risk of AMI. This means that the zero value of the event (Event = 0) reduces the risk of AMI. The fact that the event takes 1 (Event = 1) is increasing the risk of AMI (1/0.948 = 1.05 times). However, this risk is not significant (*P* > 0.05). Event meaning the mournful religious activities has not a statistically significant relationship with the number of patients with AMI per day (*P* > 0.05).

**Table 8:** Results of negative binomial Poisson regression analysis showing associations between AMI and suspected predictors

Explanatory variables	$\beta$	S.E	*P* value	RR	95% Wald C.I. for RR)	
					Lower	Upper
Event (0)^a^	− 0.053	0.088	0.551	0.948	− 0.797	1.128
Average temperature	0.011	0.004	0.014	1.011	1.002	1.019
Average humidity	0.003	0.004	0.405	1.003	0.996	1.009
Sunshine hours a day	0.025	0.007	0.001	1.025	1.010	1.040
Raining	− 0.001	0.003	0.824	0.999	0.993	1.006
Daily evaporation rate	− 0.024	0.014	0.087	0.976	0.949	1.004
Wind direction	0.003	0.001	0.097	1.000	1.000	1.002
Wind speed	0.002	0.010	0.860	1.002	0.981	1.023

^a^Event is Dummy variable, Reference’s category is 1(There is a mournful religious activity)

## Discussion

In this study, we have analysed the association between meteorological factors, emotional triggers, and epidemiological occurrence of AMI in Sari located in north of Iran during 2011–2018. It appeared that average temperature (°C) and sunshine (hour a day) are independent factors associated with an increased rate of hospital admission due to AMI during the specified period. Several studies have determined that ambient temperature is an important predictor of AMI indicating a U-shape correlation [57–60]. The findings of a systematic review and meta-analysis has shown that the association of heat exposure and heat wave is associated with increased risk of MI were immediate, whereas, the cold exposure has a delayed impact on MI incidence [61]. Contradictory findings suggest that warmer temperatures are not associated with a higher MI incidence, but the colder temperatures are associated with increased risk of MI [62]. Several underlying mechanisms are hypothesized to explain how ambient temperature is associated with increasing risk of MI that include: sympathetic nervous activity, higher blood pressure, heart rate, left ventricular end-diastolic pressure, and myocardial oxygen consumption, as well as reduced ischemia threshold, the change of hemodynamic, and coagulation [57, 63]. Furthermore, it is indicated that plaque rupture is more frequent in the colder ambient temperature that explains can cold temperatures influence on the pathogenesis of AMI [64].

Although the study has taken into account other meteorological factors such as minimum temperature, maximum temperature, wind speed, wind direction, average humidity, rain and evaporation, none had a significant impact on AMI hospital admission rates. The result yielded from the current study contradicting the findings of Madrigano et al. [7] and Radišauskas et al. [65] who showed a significant relationship between humidity, wind speed and wind pressure and AMI hospital admission. The positive association between average temperature and AMI admission is consistent with Amiya et al. [26]. Findings showing the increased in AMI incidence in summer. However, research findings in other countries have shown AMI admission in winter and spring is higher than other seasons [25, 66, 67]. According to Sharifnia et al. [68] due to geographical location of Sari (Capital of Mazandaran, Iran) a coastal city, there is a substantial change in meteorological parameters during 3 months of spring when the city undergoes a sudden rise in temperature. Higher sunshine duration was associated with higher risk of AMI. The current results provide useful information to alert health professionals to the danger of AMI incidence during this season and to take precautions when the hot season approaches.

The current study suggests an association between religious mourning and increased risk of AMI, but it was not statistically significant. Current evidences regarding the influence of religious practice on MI remains inconclusive. While some longitudinal studies that have been conducted in a large cohort of healthy people didn’t acknowledged the protective association between religious affiliation and practice [69, 70], the other study findings indicated that restful religiousness was longitudinally associated with higher odds of heart attack [71].

The context of the present study is Sari, Capital of Mazandaran state and the collected data covers the climate factors and admission numbers in this city. However, our findings can be generalized to predict the occurrence of AMI in other coastal cities with similar weather characteristics. To the best of the authors’ knowledge, the current results will be informative for people prone to AMI. This information can prepare then to take preventive measures before summer season and control the excessive emotions while attending the religious ceremonies.

### Limitation

Like many other research studies, our study is not free from limitations. Lack of access to more detailed health information of the patients such as past medical history, blood pressure and many other factors remains one of the primary limitations. Secondary, apart from meteorological and emotional factors, there are vast number of factors affecting AMI that were beyond the scope of our current research. Thirdly, the study is only limited to a specific province. Therefore, the results should be interpreted with caution.

Nevertheless, the large sample size remains as a unique feature of this study. The data for meteorological factors was extracted from the Central Station Monitoring for climate condition instead of measurements of exposure to environmental variables. This provided us the independent variables (meteorological data) that was unbiased with regards to outcome of this study.

### Recommendation

Further studies are necessary in order to quantify the emotional variables with more accurate measures. Studies with samples from different regions and also longitudinal designs are suggested to verify the findings of this study. Future studies are recommended to incorporate more detailed patients’ information about all the recognized risk factors such as BMI, blood pressure, blood cholesterol, cigarette smoking, past medical history, BUN, Creatinine.

## Conclusion

The results demonstrate that sunshine hours and average temperature had significant effects on risk of AMI.

## Data Availability

The datasets used and/or analyzed during the current study are available from the corresponding author on reasonable request.
