# Correction: A time-series prediction model of acute myocardial infarction in northern of Iran: the risk of climate change and religious mourning

**DOI:** 10.1186/s12872-022-02682-x

**Published:** 2022-05-26

**Authors:** Hamid Sharif Nia, Ozkan Gorgulu, Navaz Naghavi, Erika Sivarajan Froelicher, Fatemeh Khoshnavay Fomani, Amir Hossein Goudarzian, Saeed Pahlevan Sharif, Roghiyeh Pourkia, Ali Akbar Haghdoost

**Affiliations:** 1 Cardiovascular Research Center, Mazandaran University of Medical Sciences, Sari, Iran; 2 Department of Biostatistics and Medical Informatics, Faculty of Medicine, Ahi Evran University, Kırşehir, Turkey; 3 Faculty of Business and Law, Taylor’s University, Subang Jaya, Selangor Malaysia; 4 Department of Physiological Nursing, School of Nursing, University of California Sand Francisco, San Francisco, CA USA; 5 Department of Epidemiology and Biostatistics, School of Medicine, University of California Sand Francisco, San Francisco, CA USA; 6 School of Nursing and Midwifery, Tehran University of Medical Sciences, Tehran, Iran; 7 Student Research Committee, Mazandaran University of Medical Sciences, Sari, Iran; 8 Faculty of Business and Law, Taylor’s University Malaysia, Subang Jaya, Malaysia; 9 Department of Cardiology, Cardiovascular Research Center, Babol University of Medical Sciences, Babol, Iran; 10 Research Center for Modeling in Health, Institute for Futures Studies in Health, Kerman University of Medical Sciences, Kerman, Iran

## Correction to: BMC Cardiovascular Disorders (2021) 21:563 https://doi.org/10.1186/s12872-021-02372-0

Following publication of the original article [1], Navaz Naghavi was incorrectly denoted as the corresponding author but it should have been Fatemeh Khoshnavay Fomani.

The original article has been corrected.
